# A Novel BLNK Gene Mutation in a Four-Year-Old Child Who Presented with Late Onset of Severe Infections and High IgM Levels and Diagnosed and Followed as X-Linked Agammaglobulinemia for Two Years

**DOI:** 10.1155/2022/7313009

**Published:** 2022-06-10

**Authors:** Ezgi Topyildiz, Neslihan Edeer Karaca, Ayse Aygun, Ayca Aykut, Asude Durmaz, Guzide Aksu, Necil Kutukculer

**Affiliations:** ^1^Department of Pediatrics, Faculty of Medicine, Ege University, Izmir, Turkey; ^2^Department of Medical Genetics, Faculty of Medicine, Ege University, Izmir, Turkey

## Abstract

Agammaglobulinemia is a rare inherited immunodeficiency disorder. Mutations in the BLNK gene cause low levels of mature B lymphocytes in the peripheral blood leading to recurrent infections. We present a four-year-old Turkish boy who had recurrent respiratory tract infections in the last six months. He had very low IgG (81 mg/dl) and IgA levels (<5 mg/dl) with high IgM (258 mg/dl). Flow cytometric analysis of lymphocyte subsets showed low CD19+ B cells (0.05%). Homozygous c.790C > T (p.Gln264Ter) mutation was detected in the BLNK gene with Targeted Next Generation Sequencing (TNGS) gene analysis. Agammaglobulinemia may be due to different genetic etiologies together with complex genetic events. Although the first diagnosis to be considered in male patients is Bruton's agammaglobulinemia, patients with normal BTK sequence and/or expression should be investigated with a large genetic study such as TNGS in the early period to reach a definitive diagnosis. This male case of agammaglobulinemia highlights the necessity of considering BLNK mutations in children with B cell deficiency, even though they are known to be rare causes of agammaglobulinemia. Our case is also remarkable with high IgM levels before intravenous immunoglobulin replacement therapy and with late-onset severe infections.

## 1. Introduction

Agammaglobulinemia is a rare hereditary primary immunodeficiency disorder (PID) characterized by recurrent infections associated with low or absent circulating mature B cells and severe antibody deficiency. Approximately 85% of congenital agammaglobulinemia patients have X-linked agammaglobulinemia (XLA, Bruton's agammaglobulinemia) disease due to hemizygous mutations in the BTK gene [1, 2]. Genetic defects identified in most of the remaining patients have been shown to create an early block in B cell development. Mutations in the genes that encode components of the pre-B cell receptor (BCR) complex (IGHM, IGLL1, CD79A, CD79B) and signal transduction genes of BCR (BLNK, PIK3CD, PIK3R1, SLC39A7) cause autosomal recessive (AR) agammaglobulinemia (ARA). Recently, TOP2B mutations with autosomal dominant inheritance and TCF3 mutations with both autosomal dominant and autosomal recessive inheritance have also been identified in agammaglobulinemia cases [3, 4].

The BLNK gene (SLP-65 or BASH) encodes a 65 kDa B cell adapter molecule on chromosome 10q24.1, containing 18 exons, and plays a critical role in B lymphopoiesis [5]. While BLNK itself does not have any intrinsic enzyme activity, it functions as a scaffold for binding and assembly of molecular complexes involved in BCR-associated kinase activation. B cell signaling cascades are triggered by BLNK binding to Ig*α*, and phosphorylating by Syk on tyrosine residues that enable binding and activation of downstream signaling molecules [6]. Activated BTK and phospholipase C gamma-2 bind to the BLNK/SLP65 adaptor protein. Signaling through this complex results in the expression of the RAG proteins, which induce IgG recombination and promotes pre-B cell differentiation [5, 6].

Mutation in the BLNK gene (OMIM # 613502), first identified by Minegishi et al. in 1999 [5], had caused a failure of B cell development leading to significantly lower levels of mature B lymphocytes in the peripheral blood. These B cells cannot form plasma cells, resulting in significantly reduced levels (hypogammaglobulinemia) or absence (agammaglobulinemia) of all immunoglobulin isotypes.

Few patients with BLNK defects have been reported up to date and it is a very rare form of agammaglobulinemia [1]. Here, we report a child presenting atypically with late-onset XLA clinical findings, high IgM levels at admission, and a novel homozygous mutation in the BLNK gene detected by targeted next-generation sequencing (TNGS).

## 2. Case

A 4-year-old boy was admitted with a history of recurrent respiratory tract infections in the last six months. The parents were second-degree consanguineous and family history was unremarkable for PIDs. Physical examination was normal on admission. Initial immunologic evaluation revealed very low IgG and IgA with very high IgM levels. Flow cytometric lymphocyte subsets analysis revealed very low CD19+ B cells. Specific IgG antibodies against hepatitis A and B vaccines were both undetectable. Other laboratory tests including leukocyte, lymphocyte, haemoglobin counts, liver and kidney function tests, serologic investigations for common viruses, were all normal (Table 1). Abdominal ultrasonography revealed mild hepatosteatosis and chest radiography was normal. CD40L level was evaluated on activated T cells to exclude X-linked hiper IgM due to CD40L deficiency and it was found to be normal.

**Figure 1:**
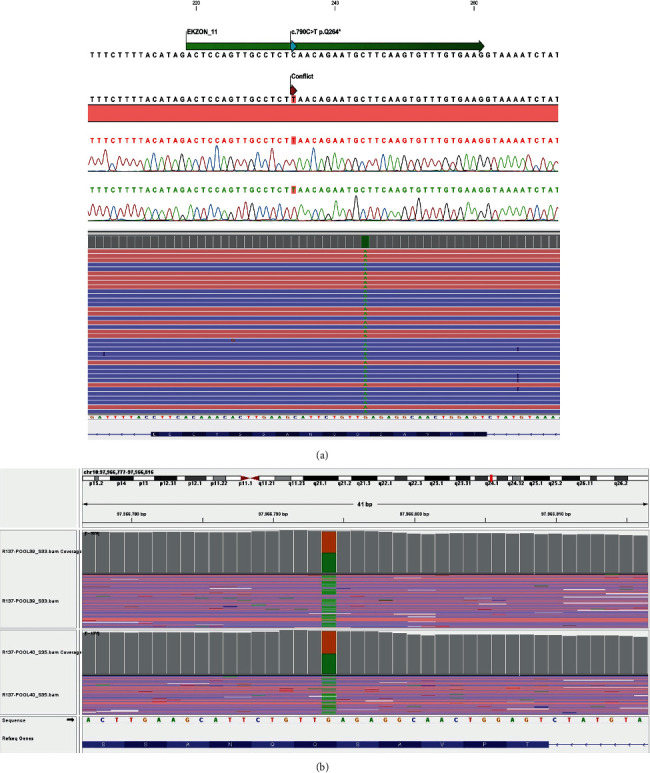
(a) Patient's BLNK gene sequence analysis images, homozygous c.790 C > T (p.Gln264Ter) mutation in exon 11. (b) His parents' heterozygous c.790 C > T (p.Gln264Ter) mutation in exon 11.

The case with recurrent respiratory infections, hypogammaglobulinemia, and decreased B cells was diagnosed as X-linked agammaglobulinemia (XLA) and intravenous immunoglobulin therapy (IVIG) (0, 4–0,8 g/kg/dose) once a month was started. Regular IVIG treatment provided IgG serum levels higher than 400 mg/dl. Targeted next-generation sequencing (TNGS) was performed in order to understand the molecular pathology two years after admission to pediatric immunology clinic. TNGS workflow based on an Ion AmpliSeq™ Primary Immune Deficiency Research Panel designed for sequencing 264 PID genes on Ion S5™ Sequencer demonstrated a homozygous c.790 C >T (p.Gln264Ter) mutation in BLNK gene (Figure 1). This novel BLNK mutation was confirmed by Sanger sequencing. Analysis of familial segregation revealed that his mother and father were heterozygous for the mutation. The patient's parents were healthy and had normal concentrations of serum immunoglobulins and normal number of B cells.

The clinical findings of this patient are similar to typical XLA patients and he was followed for two years with XLA diagnosis before TNGS gene analysis. Agammaglobulinemia was detected at 4 years of age and AR agammaglobulinemia (BLNK defect) was diagnosed at 6 years of age. The patient has been treated with IVIG replacements for 4 years, and an excellent response was obtained. To our knowledge, this patient with BLNK mutation is the ninth case in the literature and the first case to be published from Turkey. The patient is doing very well now and no major infectious episodes and autoimmune manifestations were noted up to date.

## 3. Discussion

Herein, we present a male patient who admitted with typical XLA clinical findings and was found to have a novel, unidentified, homozygous mutation (c.790C > T, p.Gln264Ter) in the BLNK gene. Eight patients with different BLNK gene defects have been published previously. Five of them had homozygous, one patient had heterozygous, and two patients had compound heterozygous mutations. As far as we know, our patient is the 9th patient with BLNK mutation reported in the literature and is the first patient reported from Turkey [5, 7–11] (Table 2). Two of the previously reported cases were Chinese, two were Arab, and two were Turkish. One of the Turkish patients is living in Europe and the second one is being followed up in a university pediatric clinic in Turkey (2nd case in Table 2) (unpublished data). One patient's ethnicity could not be learned and the last one was Scottish.

Children with autosomal recessive agammaglobulinemia present with recurrent bacterial infections, the absence of circulating B cells, and very severe hypogammaglobulinemia similar to XLA. Patients are usually symptomatic in the first year of life, after the disappearance of maternal IgG, by an average of 6 months. Autosomal recessive forms of agammaglobulinemia have been shown to present a more severe phenotype and earlier onset compared to XLA [2, 7]. However, our patient, who was asymptomatic until the age of 3.5, was diagnosed with agammaglobulinemia at the age of 4 and was followed up with a relatively mild clinical course. The late onset of infections in this case is thought to be due to being the first child of the family and very sterile life in the infantile period.

It is known that BLNK defects affect B cell development, leading to low levels of mature B lymphocytes in peripheral blood; therefore plasma cells cannot be formed, thus causing a significant decrease in all immunoglobulin isotypes. However, there is no detailed information about the effect of BLNK defect on T cell development. A Chinese patient with BLNK defect was reported to have lower memory CD3+CD4+ and CD3+CD8+ T cells than healthy controls, whereas naive T cells were higher levels than controls [11]. Although the cases generally present with low immunoglobulin values and low circulating B cell numbers, a 28-year-old BLNK patient with only low IgM was reported [10]. Contrary to this finding, the IgM level in our patient's first admission was found to be high as compared to age-related normal levels. Our patient was the first patient admitted with high IgM in the literature. After the control and management of infections under regular IVIG treatment, the patient's IgM value decreased to undetectable values during follow-up (Table 3).

Most of the patients with BLNK defect presented with bacterial respiratory tract infections. To prevent these infections, patients with agammaglobulinemia need lifelong immunoglobulin replacement therapy. Since immunoglobulin replacement reduces life-threatening infections, patients reach adulthood with regular treatment and follow-up, and quality of life of almost all of these cases is very good [12]. However, severe infections can also be observed in some cases. For example, systemic enteroviral infections have been reported in patients previously diagnosed with agammaglobulinemia. One child with Ig*α* deficiency, one of the different forms of ARA, had polio due to wild-type vaccine at the age of 12 months (M.E. Conley and V. Howard, unpublished observations), while another child had typical signs of enteroviral infection such as weakness and dermatomyositis-like syndrome [13]. Another patient reported by NaserEddin et al. showed skin and joint findings as described in patients with BTK deficiency despite regular IVIG therapy, and the patient's peripheral blood was simultaneously found to be positive for enterovirus shown by polymerase-chain reaction [8]. As a different finding, it has been reported that neutropenia may also accompany BLNK deficiency. A patient with agammaglobulinemia and neutropenia was reported but exact diagnosis could not be established because of his death due to *Pseudomonas* sepsis at the age of 16 months, and this patient's sibling was diagnosed as BLNK deficiency after a few years. Contrary to these reported cases, our patient did not have so much serious infections and neutropenia was not observed. He is now living without severe infections and complications under regular IVIG replacement therapy.

## 4. Conclusion

Agammaglobulinemia may be due to different etiologies with complex genetic events. XLA is the first diagnosis to be considered in male cases. Patients with normal BTK sequence should be investigated with a broad-spectrum genetic study for an exact and early diagnosis. This case of agammaglobulinemia in a 4-year-old male patient highlights a novel autosomal recessive mutation in BLNK gene. Although recessive BLNK mutations appear to be rare causes of agammaglobulinemia (given the small number of reported cases), they should be considered in B-cell-deficient children. While our presented child remains stable on monthly immunoglobulin therapy, long-term follow-up will be required to determine the outcome of this mutation and other health outcomes, given the lack of published literature on individuals with recessive BLNK mutations.

## Figures and Tables

**Table 1:** Immunologic evaluations at admission and age-related normal levels.

	Patients value	Normal range
Leukocyte (10^3^/*μ*L)	11,40	5–13
Neutrophil (10^3^/*μ*L)	4, 25	2–6, 9
Lymphocyte (10^3^/*μ*L)	5, 26	1, 5–3, 4
Haemoglobin (g/dl)	11, 86	12–15
Thrombocyte (10^3^/*μ*L)	483	142–424
IgG (mg/dl)	81	894 ± 157
IgM (mg/dl)	258	92 ± 35
IgA (mg/dl)	<5	72 ± 22
Total IgE (IU/ml)	4, 1	2–307
CD3+ T cells ((%)/*μ*L)	92, 1/4844	55–79/1900–3600
CD19+ B cells ((%)/*μ*L)	0,05/2,6	11–31/300–1200
CD3+CD4+ T cells ((%)/*μ*L)	59, 3/3119	26–49/600–2000
CD3+CD8+ T cells ((%)/*μ*L)	29, 3/1541	9–35/300–1300
CD3-CD16+CD56+ NK cells ((%)/*μ*L)	5, 7/299	5–28/200–1200
Anti-HBs (mIU/ml)	Negative	10–1000
Anti-HAV IgG	Negative	>0, 9
Anti-CMV IgG (aU/ml)	Negative	6–250

**Table 2:** Reported patients with BLNK protein deficiency.


Patient (reference)	Age at onset (years), gender, ethnicity	Infection profile	Additional manifestations	Ig levels at diagnosis (mg/dL)	Circulating CD19^+^ B cells (%)	BLNK Mutation
Patient 1 (Minegishi et al. [5])	8 months, male, Caucasian of Scot/Irish ancestry	Recurrent otitis and pneumonia hepatitis C	Intermittent protein losing enteropathy	Undetectable	0.05	Homozygous c.30°C > A (p.P10P)/c.47 + 3 A > T
Patient 2 (Conley et al. [2])	8, female, Turkish	Recurrent respiratory infections, diarrhea, otitis, septic arthritis, and conjunctivitis	Resolved hepatitis with no clear diagnosis	IgG 111 IgA < 6 IgM 10	0.01	Homozygous c.367 C > T (p.R123X)
Patient 3 (Lagresle-Peyrou et al. [9])	6, male, NA	Recurrent otitis and pneumonia	None	Undetectable	0	Homozygous c.844 C > T (p.R282X)
Patient 4 (NaserEddin et al. [8])	0.5, male, Arab	Recurrent otitis media, chronic diarrhea, enteroviral viremia	Chronic polyarthritis, dermatitis and sensorineural hearing loss	Undetectable	0	Homozygous c.435_436 del T CInsA (p.E145fs25*∗*)
Patient 5 (NaserEddin et al. [8])	1, female (elder sister of P4), Arab	Recurrent diarrhea, otitis media and sino-pulmonary infections	Arthritis, bronchiectasis	Undetectable	0	Homozygous c.435_436 del T CInsA (p.E145fs25*∗*)
Patient 6 (Geier et al. [10])	28, male, Turkish	No increased susceptibility to infections	Chronic renal insufficiency	IgG 903 IgA 791 IgM 27 (selective IgM deficiency)	14	Compound heterozygous c328 C > G (pPro110Ala)/c472 G > T (pAla158Ser)
Patient 7 (Niu Li et al. [11])	5, female, Chinese	Respiratory infections, including sinusitis, bronchitis, and pneumonia	Epilepsy, allergic rhinitis and wheezing	IgG 135 IgA < 6 IgM < 18	3.5	Compound heterozygous c.676 + 1 G > A, exon 9 deletion, c.677_746del, p.R227Kfs *∗* 7
Patient 8 (Niu Li et al. [11])	2, male, Chinese	Recurrent bronchitis, pneumonia, and acute lymphadenitis	None	Undetectable	3	Heterozygous frameshift variant c.452_453dup CC, (p.T152Pfs *∗* 6), c. 525G > A
Patient 9 (our presented case)	3.5, male, Turkish	Recurrent respiratory tract	None	IgG 81 IgA < 5 IgM 258	0,05	Homozygous mutation c.790 C > T (p.Gln264Ter)

**Table 3:** Patient's Ig levels by age.

	Initial visit	Second visit	5-year-old	6-year-old	7-year-old	8-year-old
IgG (mg/dl)	81 (*n*: 894 ± 157)	134 (*n*: 894 ± 157)	422 (*n*: 1008 ± 209)	423 (*n*: 1008 ± 209)	569 (*n*: 1061 ± 203)	725 (*n*: 1061 ± 203)
IgM (mg/dl)	258 (*n*: 92 ± 35)	131 (*n*: 92 ± 35)	18, 4 (*n*: 113 ± 40)	<17 (*n*: 113 ± 40)	<17 (*n*: 106 ± 43)	<19 (*n*: 106 ± 43)
IgA (mg/dl)	<5 (*n*: 72 ± 22)	6,6 (*n*: 72 ± 22)	6, 5 (*n*: 99 ± 37)	6, 5 (*n*: 99 ± 37)	<27 (*n*: 116 ± 42)	<28 (*n*: 116 ± 42)
Treatment	—	Initiation of IVIG therapy	IVIG therapy (IVIG) (0, 5 g/kg/dose) once a month			

## Data Availability

The data used to support the findings of this case report are included within the article.
